# Intraosseous blood samples for point-of-care analysis: agreement between intraosseous and arterial analyses

**DOI:** 10.1186/s13049-017-0435-4

**Published:** 2017-09-11

**Authors:** Milla Jousi, Simo Saikko, Jouni Nurmi

**Affiliations:** 1 Emergency Medicine and Services, Helsinki University Hospital and Department of Emergency Medicine, University of Helsinki, Finland, HYKS Akuutti, PL 340, 00029 HUS Helsinki, Finland; 2 Saimaa University of Applied Sciences, Skinnarilankatu 36, 53850 Lappeenranta, Finland; 3 FinnHEMS Research and Development Unit, Lentäjäntie 3, 01530 Vantaa, Finland

**Keywords:** Point-of-care, Blood gas analysis, Intraosseous access, I-STAT

## Abstract

**Background:**

Point-of-care (POC) testing is highly useful when treating critically ill patients. In case of difficult vascular access, the intraosseous (IO) route is commonly used, and blood is aspirated to confirm the correct position of the IO-needle. Thus, IO blood samples could be easily accessed for POC analyses in emergency situations. The aim of this study was to determine whether IO values agree sufficiently with arterial values to be used for clinical decision making.

**Methods:**

Two samples of IO blood were drawn from 31 healthy volunteers and compared with arterial samples. The samples were analysed for sodium, potassium, ionized calcium, glucose, haemoglobin, haematocrit, pH, blood gases, base excess, bicarbonate, and lactate using the i-STAT® POC device. Agreement and reliability were estimated by using the Bland-Altman method and intraclass correlation coefficient calculations.

**Results:**

Good agreement was evident between the IO and arterial samples for pH, glucose, and lactate. Potassium levels were clearly higher in the IO samples than those from arterial blood. Base excess and bicarbonate were slightly higher, and sodium and ionised calcium values were slightly lower, in the IO samples compared with the arterial values. The blood gases in the IO samples were between arterial and venous values. Haemoglobin and haematocrit showed remarkable variation in agreement.

**Discussion:**

POC diagnostics of IO blood can be a useful tool to guide treatment in critical emergency care. Seeking out the reversible causes of cardiac arrest or assessing the severity of shock are examples of situations in which obtaining vascular access and blood samples can be difficult, though information about the electrolytes, acid-base balance, and lactate could guide clinical decision making.

The analysis of IO samples should though be limited to situations in which no other option is available, and the results should be interpreted with caution, because there is not yet enough scientific evidence regarding the agreement of IO and arterial results among unstable patients.

**Conclusions:**

IO blood samples are suitable for analysis with the i-STAT® point-of-care device in emergency care. The aspirate used to confirm the correct placement of the IO needle can also be used for analysis. The results must be interpreted within a clinical context while taking the magnitude and direction of bias into account.

## Background

Point-of-care (POC) laboratory diagnostics can be used to screen for critical conditions and guide treatment in pre-hospital emergency care. Resuscitation guidelines recommend the review and correction of potentially reversible causes of cardiac arrest during resuscitation [1], and POC testing can sometimes add information. Many physician- or paramedic-based pre-hospital emergency medical services (EMS) use handheld POC analysers. Analyses are usually made from arterial or venous blood. However, drawing a sample from a critically ill patient can be challenging due to haemodynamic collapse or cold environments – the same factors responsible for difficulties with vascular access.

The intraosseous (IO) route is recommended for medication when intravenous access is difficult or impossible [1]. As a result of the availability of feasible power-driven IO devices, IO access has become widely used for vascular access during cardio-pulmonary resuscitation (CPR) and other critical conditions. Normally, a small amount of bone marrow is aspirated via the IO needle after insertion to confirm correct needle placement. This aspirate could be made rapidly available for POC diagnostics.

It is unclear, how the results of IO blood sample analyses should be interpreted and how exactly the parameters agree with venous or arterial values because evidence regarding the laboratory analysis of IO samples is controversial. Several animal and human studies have been published investigating different laboratory parameters using both conventional laboratory and POC devices for the analyses [2–19]. Because most of the previous studies [5, 8, 13, 18] have evaluated the correlations between two methods instead of their agreement [20], better evaluations of agreement and bias are needed before IO samples can be used for clinical decision making.

The aim of this study was to determine whether the intraosseous POC values of parameters that are commonly used in emergency diagnostics agree with the arterial values with sufficient precision to allow the POC values of IO samples to be used for clinical decision making in emergency situations.

## Methods

### Study design

We conducted an observational study comparing the POC values of IO blood samples to those of venous and arterial blood samples from 31 healthy volunteers. The research was conducted according to the principles of the Declaration of Helsinki, and the study protocol was approved by the Coordinating Ethics Committee of Helsinki University Hospital (ref no 250/13/03/00/15). This study was investigator-initiated and carried out without a sponsor. Medidyne Oy, Finland, provided equipment for IO accesses.

### Study setting

Data were collected during a training session in which paramedic students practiced inserting an IO needle on each other under supervision. Healthy volunteers aged over 18 years were recruited for the study. Exclusion criteria were skin infection around the puncture site, immunocompromising condition or medication, pregnancy, and breast-feeding. Written informed consent was obtained from all the participants, and follow-up was organised in cases of complications.

We used the EZ-IO® device (Teleflex®, Inc., PA, U.S.A.) with a 15G 25-mm needle to obtain IO access to the proximal tibia by using a sterile technique involving facial masks, sterile gloves, and surgical skin disinfection with alcohol. We drew two IO samples for analysis from each participant to evaluate the need for waste blood before actual sampling: the initial sample was 0.5 ml (IO1), followed by a second sample after the removal of 2 ml of waste blood (IO2). Immediately after these, we took blood samples from the antecubital vein and the radial artery. We drew all the samples with dry heparin (70 IU, 3 ml) blood gas syringes (RAPIDLyte®, Siemens Healthcare Diagnostics GmbH®, Erlangen, Germany). If we encountered difficulties drawing the IO sample with the dry heparin syringe, we used a normal 2-ml syringe for aspiration and then immediately injected the sample into a heparinised syringe. We analysed all blood samples with a point-of-care device (i-STAT® handheld, Abbott Point of Care, Inc., NJ, U.S.A.) and used CG8 + −cartridges for the analyses of sodium (Na), potassium (K), ionised calcium (iCa), glucose (Gluc), haemoglobin (Hb), haematocrit (Hct), pH, partial pressure of carbon dioxide (pCO_2_), partial pressure of oxygen (pO_2_), base excess (BE), and standard bicarbonate (HCO_3_). We used CG4 + −cartridges for the analyses of lactate (Lact).

We compared the POC values analysed from two intraosseous blood samples (IO1 and IO2) to POC values analysed from arterial blood. Additionally, we compared venous samples to arterial samples as a reference, and we compared the IO1 values to IO2 values to assess the effect of drawing waste blood.

### Statistical analysis

We used the Bland-Altman method [20–23] to calculate bias (with 95% confidence interval [CI]) and limits of agreement, and we drew the graphs for each parameter using GraphPad Prism version 7.0a (GraphPad Software, Inc., California, U.S.A.) and R version 3.3.1 (The R Foundation for Statistical Computing, Vienna, Austria)**.** For proportions, 95% CI was calculated using the modified Wald method. We calculated intraclass correlation coefficients (ICC) with 95% confidence intervals [CI] to assess the reliability of the measurements by using IBM SPSS Statistics version 24 (IBM Corporation, NY, U.S.A.) based on a single measurement, consistency, 2-way mixed-effects model. Sample size calculation was based on achieving clinically sufficient confidence intervals for the bias and the limits of agreement.

The Bland-Altman method is an illustrative statistical method for assessing the agreement between two clinical measurement methods [20]. It yields an informative graph regarding the agreement in which the individual differences in the results that are measured using the two different methods are plotted against the means of the measurements to display the average bias and the limits of agreement. In this case, the bias represents systematic error, and the limits of agreement represent random error describing the variation of the differences. The intraclass correlation coefficient (ICC) is a reliability index usually used in test-retest, intrarater, and interrater reliability analyses.

## Results

**Fig. 1:**
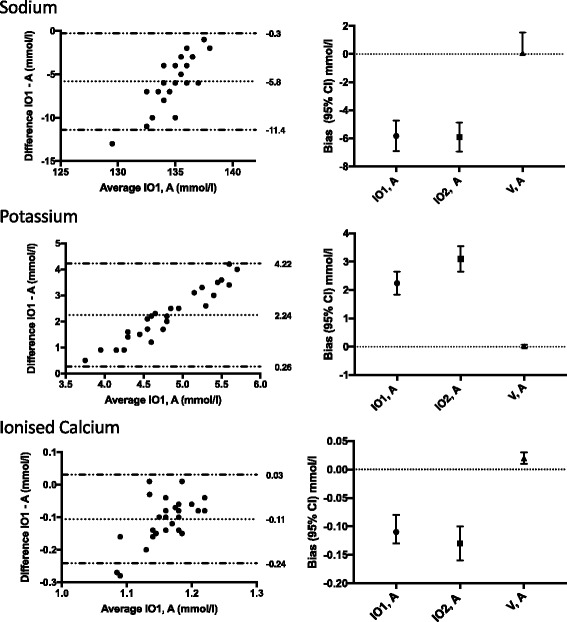
Bland-Altman graphs of intraosseous and arterial samples (left panels), and the comparison of the biases between intraosseous samples and venous samples in reference to arterial samples (right panels) for POC measurements of potassium, sodium, and ionised calcium. The dotted lines on the Bland-Altman graphs indicate bias and the limits of agreement (+/− 1.96 SD). The error bars on the right panels represent biases (95% confidence intervals). IO1 – first intraosseous sample; IO2 – second intraosseous sample after 2 ml of waste blood was drawn; V – venous sample; A – arterial sample

**Fig. 2:**
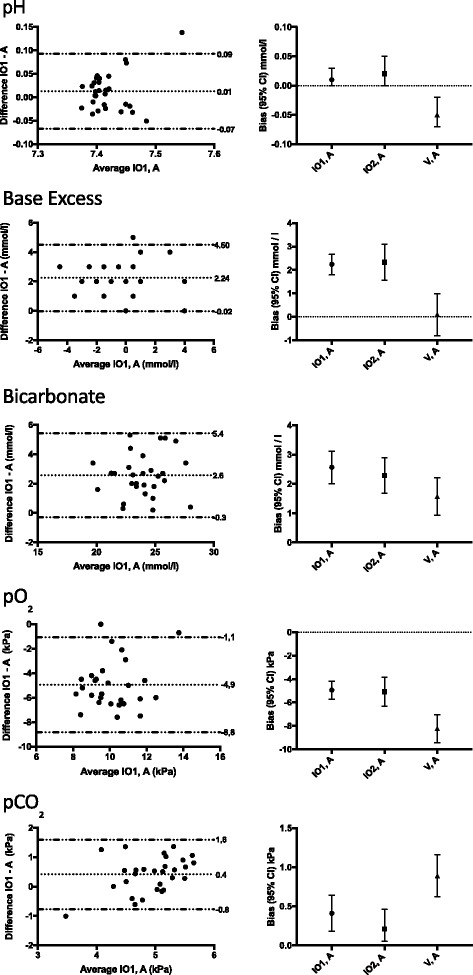
Bland-Altman graphs of intraosseous and arterial samples (left panels), and the comparison of the biases between intraosseous samples and venous samples in reference to arterial samples (right panels) for POC measurements of pH, base excess, bicarbonate, pO_2_, and pCO_2_. The dotted lines on the Bland-Altman graphs indicate bias and the limits of agreement (+/− 1.96 SD). The error bars on the right panels represent biases (95% confidence intervals). IO1 – first intraosseous sample; IO2 – second intraosseous sample after 2 ml of waste blood was drawn; V – venous sample; A – arterial sample

**Fig. 3:**
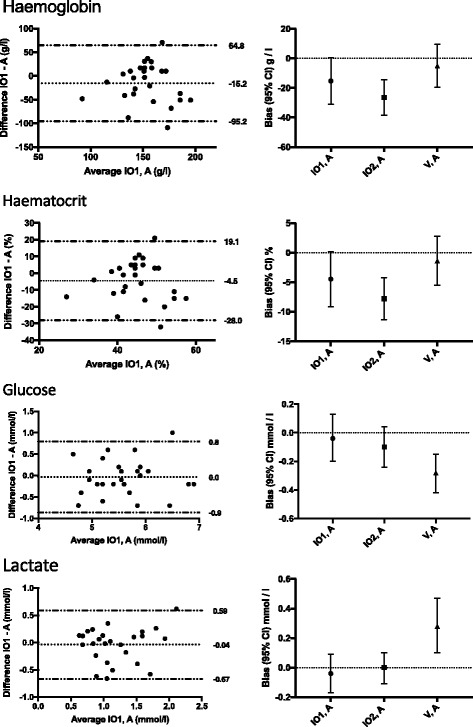
Bland-Altman graphs of intraosseous and arterial samples (left panels), and the comparison of the biases between the intraosseous samples and venous samples in reference to arterial samples (right panels) for POC measurements of haemoglobin, haematocrit, glucose, and lactate. The dotted lines on the Bland-Altman graphs indicate bias and the limits of agreement (+/− 1.96 SD). The error bars on right panels represent biases (95% confidence intervals). IO1 – first intraosseous sample; IO2 – second intraosseous sample after 2 ml of waste blood was drawn; V – venous sample; A – arterial sample

The mean age of the 31 participants was 25 years (range 19–36, SD 4.1). Thirteen (42%) of the participants were men. In 7 (23%, 95% CI 11 to 40%) cases, the aspiration of IO blood was difficult and more time and/or power was required to aspirate the sample. The POC analysis of IO blood was successful in 90% (95% CI 74 to 97%) of the cases (IO1 CG8+ 94%, IO1 CG4+ 84%, IO2 CG8+ 97%, IO2 CG4+ 84%). Unsuccessful analyses were due to an insufficient amount of aspirate and/or clotting of the sample. An overview of the POC measurement values is presented in Table 1. Great variation was observed within the biases for the analysed parameters between IO1 and the arterial samples (Figs. 1, 2 and 3).

**Table 1:** The point-of-care results for the analyses of IO1 (initial intraosseous sample), IO2 (second intraosseous sample after 2 ml of waste blood), arterial blood, and venous blood

	IO1	IO2	Artery	Vein
Na (mmol/l)	132 (123–137) *n* = 29	131.5 (125–138) *n* = 30	138 (131–140)	138 (132–142)
K (mmol/l)	5.8 (4.0–7.7) *n* = 27	6.9 (4.5–8.8) *n* = 26	3.7 (3.5–4.2)	3.7 (3.3–4.2)
iCa (mmol/l)	1.12 (0.95–1.2) *n* = 29	1.07 (0.91–1.24) *n* = 30	1.21 (1.13–1.26)	1.23 (1.18–1.3)
pH	7.420 (7.363–7.614) n = 29	7.433 (7.379–7.679) *n* = 30	7.408 (7.356–7.789)	7.377 (7.298–7.463)
BE (mmol/l)	1.0 (−3.0–5.0) *n* = 29	1.0 (−3.0–6.0) *n* = 30	−1.0 (−6.0–9.0)	−1.0 (−5.0–4.0)
HCO_3_ (mmol/l)	25.1 (20.9–29.3) *n* = 29	25.0 (20.7–30.6) *n* = 30	22.6 (18.0–27.8)	24.4 (20.8–28.7)
pO_2_ (kPa)	7.35 (4.7–13.4) *n* = 28	7.55 (4.1–13.7) *n* = 30	12.35 (9.5–15.5) *n* = 30	4.3 (2.6–10.5)
pCO_2_ (kPa)	5.12 (2.97–6.16) *n* = 29	4.96 (2.44–6.16) *n* = 30	4.82 (2.25–5.36)	5.55 (4.07–7.29)
Hb (g/l)	153 (68–204) *n* = 28	139.5 (92–190) *n* = 30	153 (116–228)	150 (119–241) *n* = 30
Hct (%)	45 (20–60) *n* = 28	41 (27–56) *n* = 30	45 (34–67)	44 (35–71)
Gluc (mmol/l)	5.5 (4.4–7.0) *n* = 27	5.5 (4.6–6.8) *n* = 29	5.5 (4.4–7.0)	5.4 (3.8–6.6)
Lact (mmol/l)	1.03 (0.58–2.42) *n* = 26	1.04 (0.68–2.11) *n* = 26	1.15 (0.56–2.0) *n* = 30	1.44 (0.74–2.58)

Data are presented as median (range). The number of results is indicated only if it differed from 31. Potassium values over 9.0 mmol/l were excluded from the analysis as outliers (the upper limit of measurement for the used method, IO1 *n* = 2, IO2 *n* = 4)

Regarding electrolytes, the potassium values were 2.2 mmol/l and 3.1 mmol/l higher compared with those for arterial blood for IO1 and IO2, respectively. The sodium and ionised calcium values were slightly lower in the IO1 samples than in arterial samples (Fig. 1).

The analyses of the IO samples showed higher results for base excess and bicarbonate compared with the arterial values, whereas for pH, there was good agreement (Fig. 2). The partial pressures of oxygen and carbon dioxide in the IO samples fell between the arterial and venous pressure values, which is physiologically explicable.

For haemoglobin and haematocrit, the IO results showed remarkable variation in agreement compared with the arterial samples (Fig. 3).

For the glucose and lactate values, there was good agreement between IO1 and arterial samples (Fig. 3).

Good agreement between IO1 and IO2 results was found for all parameters except K and Hb (Table 2).

**Table 2:** Bias (95% confidence interval) between the IO1 (initial) and IO2 (after 2 ml of waste blood) results, calculated using the Bland-Altman method

Na (mmol/l)	−0.14 (−1.17; 0.88)
K (mmol/l)	−0.77 (−1.17; −0.38)
iCa (mmol/l)	0.02 (−0.01; 0.05)
pH	−0.02 (−0.03; −0.01)
BE (mmol/l)	−0.32 (−0.69; 0.04)
HCO_3_ (mmol/l)	0.2 (−0.15; 0.55)
pO_2_ (kPa)	−0.26 (−1.06; 0.54)
pCO_2_ (kPa)	0.25 (0.11; 0.39)
Hb (g/l)	11.52 (−3.69; 26.72)
Hct (%)	3.41 (−1.07; 7.89)
Gluc (mmol/l)	0.07 (0; 0.13)
Lact (mmol/l)	−0.05 (−0.12; 0.01)

Intraclass correlation coefficient (ICC) calculations suggest moderate to good reliability for lactate, glucose, BE and HCO_3_ measurements when the IO1 values were compared with the arterial values (Table 3).

**Table 3:** Intraclass correlation coefficients with 95% confidence intervals (a single measurement, consistency, 2-way mixed-effects model) for IO1 versus arterial sample results

	ICC (95% CI)
Na (mmol/l)	0.174 (−0.200; 0.503)
K (mmol/l)	0.050 (−0.317; 0.404)
iCa (mmol/l)	−0.004^a^ (−0.364; 0.358)
pH	0.513 (0.187; 0.738)
BE (mmol/l)	0.837 (0.682; 0.738)
HCO_3_ (mmol/l)	0.765 (0.559; 0.883)
pO_2_ (kPa)	0.282 (−0.095; 0.588)
pCO_2_ (kPa)	0.460 (0.119; 0.704)
Hb (g/l)	0.104 (−0.274; 0.454)
Hct (%)	0.100 (−0.277; 0.451)
Gluc (mmol/l)	0.779 (0.571; 0.893)
Lact (mmol/l)	0.738 (0.497; 0.873)

^a^ ICC is negative due to low between-subjects variance

## Discussion

This study shows that POC diagnostics can be performed using samples obtained during the insertion of the IO needle. As no significant difference was observed in the results with or without drawing waste blood before sampling, it seems feasible to analyse blood that is initially drawn to confirm the position of the needle. We found significant variation in the agreement between the results from the arterial and IO samples. Thus, clinical consideration and awareness of bias is needed for interpreting the POC results from an IO sample.

Based on the results of this study, glucose, lactate, and pH results of IO POC samples are suitable to consider for decision making. PCO_2_ values might be used with the understanding that the IO value is moderately higher than the arterial value. Furthermore, pO_2_ results might be used to exclude hypoxia if data from the pulse oximeter is unavailable, acknowledging that the IO value is lower than the arterial. BE and HCO_3_ could probably be used, as well as ionised calcium to exclude severe hypocalcaemia, and sodium could be used to exclude severe hyponatraemia. The use of IO POC values of potassium, haematocrit, and haemoglobin for decision making cannot be recommended.

POC diagnostics of intraosseous blood can be a useful tool to guide treatment in critical emergency care. Seeking out the reversible causes of cardiac arrest or assessing the severity of shock are examples of situations in which obtaining vascular access and blood samples can be difficult, though information about the electrolytes, acid-base balance, and lactate could guide clinical decision making.

There are several earlier studies comparing IO blood to either arterial or venous blood in laboratory analysis [2–19]; however, their evidence is controversial, predominantly due to varying statistical methods and small sample sizes. In most studies, the conclusions were drawn from Pearson’s correlation coefficients, linear regression, or comparisons of the grouped means of parameters. However, while correlation generally indicates a linear relationship between two measurements, it is not a measure of agreement between two methods [20]. Neither is linear regression, because it is based on correlation coefficients. Even if median or mean values of certain parameters seem to be similar for IO and arterial values, it is necessary to compare the differences within individuals, not the mean/median values of whole study populations. Agreement, which is a much more relevant clinical measurement, has been evaluated in a few studies [4, 7, 10, 11, 14, 15]. The results concerning different laboratory parameters vary among these studies. In agreement with our study, there appears to be a consensus that intraosseous potassium values are uniformly higher than arterial/venous values.

The tolerance of bias and minimum precision always depends on the actual clinical situation, the parameter, and the actual value of the parameter. Therefore, it is impossible to categorise the reliability of the IO POC results. The decision, whether the bias and precision are acceptable for the actual situation of a particular patient, must be left to the clinician.

The analysis of IO samples should be limited to situations in which no other option is available, and the results should be interpreted with caution, because there is not yet enough scientific evidence regarding the agreement of IO and arterial results among unstable patients.

In this study, all samples were drawn from healthy young people and represent a normal range of values. Circulation in the bone marrow cavity is compromised during cardiac arrest, cardiopulmonary resuscitation, and shock. Centralisation of the circulation to the vital organs and hypoperfusion or stasis in the bone marrow cavity may significantly affect the IO results. Agreement during critical illness might be very different from our results, and the results here cannot be extrapolated to different patient groups. There could also be variation among different IO access sites (proximal humerus or proximal tibia) and in different age groups as the amount of red marrow decreases with age and persists longer in the proximal humerus.

The remarkable variation in agreement of the haemoglobin and haematocrit values in our study indicates a random rather than systematic error within the measurements. This random error might be caused by haemolysis during sample aspiration and/or clotting of the samples or by the haematopoietic capacity of young students’ red bone marrow in the proximal end of the tibia.

The bias between IO and arterial results might be partly caused by artefacts in the sampling process, such as haemolysis, clotting, or incomplete removal of air from the syringe. In our opinion, these represent real life scenarios, which paramedics and emergency doctors might encounter when drawing and analysing IO or arterial samples. The impact of single measurement errors on the overall results of this study must also be considered because of the relatively small sample size.

We used intraclass correlation coefficients to analyse the reliability of the measurements. In interpreting the ICC results, one should keep in mind that a low ICC reflects not only low agreement among measurements but also the lack of variability among test results [24–26].

Further studies with critically ill patients could yield important knowledge, whether our findings persist in a wider range of values and in different haemodynamic states.

## Conclusions

Intraosseous blood samples are suitable for analysis with the i-STAT® point-of-care analyser. There is no need to draw waste blood before actual sample; the aspirate used for confirming the correct placement of the IO needle can be used for analysis. The agreement between IO and arterial POC results varies for different parameters. The results must be interpreted within a clinical context taking the magnitude and direction of bias into account. The tolerance to bias and precision of the measurement is always dependent on the clinical situation.

## Abbreviations

BE: Base excess
CPR: Cardio-pulmonary resuscitation
EMS: Emergency medical service
Gluc: Glucose
Hb: Haemoglobin
HCO_3_: Standard bicarbonate
Hct: Haematocrit
iCa: Ionised calcium
ICC: Intraclass correlation coefficient
IO: Intraosseous
K: Potassium
Lact: Lactate
Na: Sodium
pCO_2_: Partial pressure of carbon dioxide
pO_2_: Partial pressure of oxygen
POC: Point-of-care
